# Supplementary material for: Genomic and Cytotoxic Damage in Wistar Rats and Their Newborns After Transplacental Exposure to Hibiscus sabdariffa Hydroalcoholic Extract
Source: Int J Mol Sci. 2025 Aug 1;26(15):7448. doi: 10.3390/ijms26157448 (PMC12347892; doi:10.3390/ijms26157448)
Supplement: Supplementary file 1 [file ijms-26-07448-s001.zip › ijms-3733372-supplementary.pdf]

SUPPLEMENTARY MATERIAL

Phytochemical characterization of the EH*Hs*

The pH of the extract was 2.2, with a yield of 40 ± 5% g/g, and it had a deep red color (Figure S1).

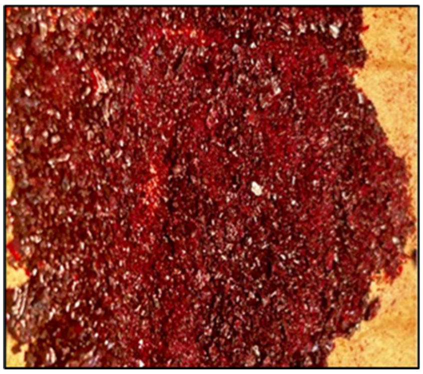

Figure S1 Hydroalcoholic extract of Hibiscus sabdariffa

Total anthocyanin content

As part of the characterization of the hydroalcoholic extract of *Hibiscus sabdariffa* (EH*Hs*), the presence of anthocyanins was confirmed using the pH differential method. This technique quantifies total anthocyanin content based on the reversible structural changes of the anthocyanin chromophore, which shifts from the colored oxonium form (pH 1.0) to the hemiketal form at pH 4.5.

UV-Vis spectrophotometry aids in identifying anthocyanins, which display a characteristic absorption pattern: two bands in the UV region at 260–280 nm and another at 310–340 nm. The latter is associated with acylation of the molecule, and its absence or appearance as a shoulder suggests the presence of non-acylated anthocyanins. A second maximum is observed in the visible region between 490 and 550 nm, with a shoulder between 400 and 450 nm, whose intensity depends on the number of sugar moieties attached to the aglycone.

The anthocyanin content was calculated according to the method described by *Lee J. et al. (2005),* using absorbance readings at 520 nm and 700 nm at both pH values, along with the molecular weight and extinction coefficient of cyanidin-3-glucoside. The resulting content was 564.42 mg of cyanidin-3-glucoside equivalents per 100 g of extract. These results are consistent with the anthocyanins previously reported in *H. sabdariffa*, mainly delphinidin and glycosylated cyanidin, both of which absorb at 520 nm. The other identified signals are

related to the number of hydroxyl groups on the B-ring of the anthocyanidin, as well as the type, number, and position of sugars attached to the aglycone, and the presence or absence of aromatic or aliphatic acids bound to the sugars (acylation).

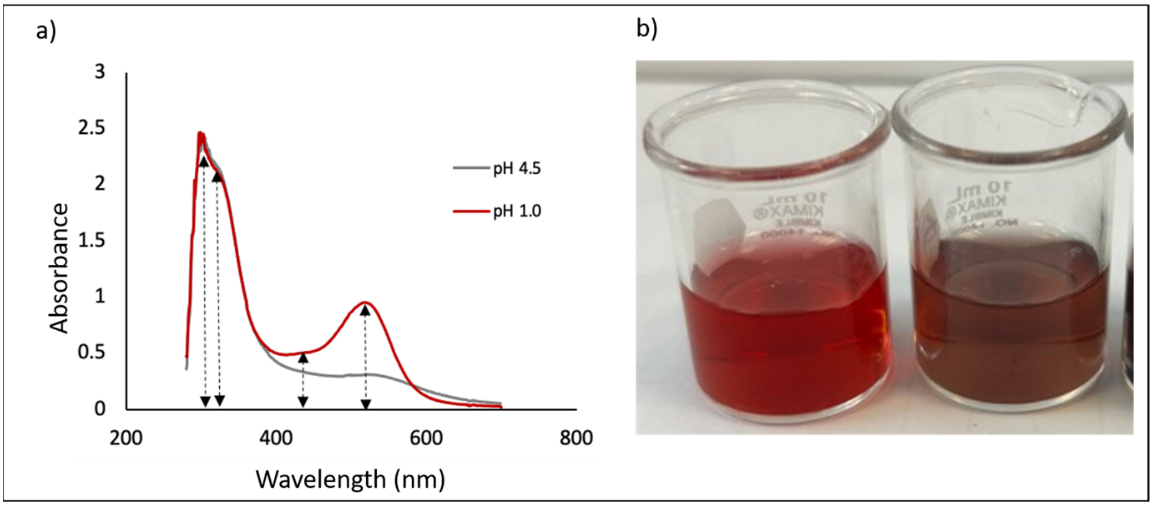

Figure S2 *EHHs analysis* ***(Figure S2 a)*** *UV-Vis spectrum of the EHHs in aqueous solution at pH 1.0 (red line) and pH 4.5 (gray line), along with their corresponding colors (left: pH 1.0, right: pH 4.5). At pH 1.0, maximum absorption was identified in the visible region at 520 nm and in the UV region at 300 nm, along with two shoulders, one around 320 nm and another, less prominent, around 430 nm. At pH 4.5, a significant decrease in absorbance at 520 nm was observed* ***(Figure S2 b).*** *The color of the solutions shifted from a bright red at pH 1.0 to a reddish-brown at pH 4.5 (Figure 2 b). At pH 7.0 (not shown), the solution displayed a blue color corresponding to the quinonoidal base.*

Total phenolic content

Other important compounds in *H. sabdariffa* associated with its biological activity include phenolic compounds, organic acids, and flavonoids. In this study, we quantified the total phenolic content using the Folin-Ciocalteu (FC) method. The FC reagent is a complex mixture of phosphomolybdic and phosphotungstic acids, which are reduced during the electron transfer from reducing compounds in the sample to the FC reagent, forming a blue chromophore with maximum absorbance at 760 nm. Gallic acid was used as the reference compound to generate a calibration curve (Figure S2 a), from which the total phenolic content in the EH*Hs* was determined to be 4.82 g of gallic acid equivalents per 100 g of extract.

Total flavonoid content

Similarly, a colorimetric method was used to quantify the flavonoid content in the EH*Hs*. This method involves aluminum chloride, which forms stable complexes under acidic conditions with the ketone group at C4 of flavones or with the hydroxyl groups at C3 or C5

in flavonols. Additionally, the reagent may react with ortho-dihydroxyl groups on rings A or B of flavonoids, forming labile complexes in acidic solutions. In both cases, the resulting complexes produce a pink coloration measured at 510 nm.

Rutin was used as the reference standard to construct a calibration curve (Figure S2 b). Based on this, the total flavonoid content in the EH*Hs* was determined to be 2.62 g of rutin equivalents per 100 g of extract.

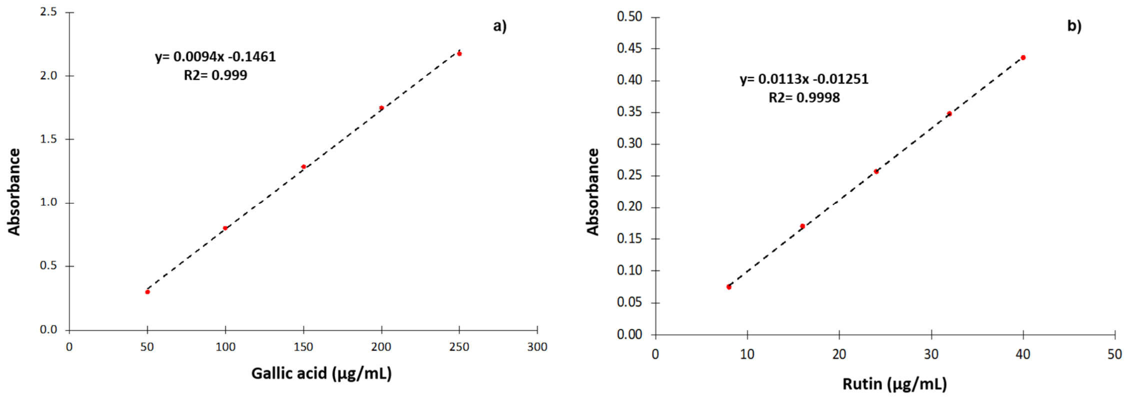

Figure S3 *Calibration curves for the quantification of* ***(Figure S3 a)*** *total phenolic compounds and* ***(Figure S3 b)*** *flavonoids in the EHHs. Each point represents the average of three independent measurements ± SD.*

Gas chromatography–mass spectrometry

Secondary metabolites present in the EH*Hs* were identified using a gas chromatography–mass spectrometry (GC-MS) system (Agilent 7890B-5977A-MSD). The acquisition software used was MassHunter GC/MS Acquisition, version B.07.02.1938. Separation was performed on an HP-5ms capillary column (30.0 m length, 0.25 mm diameter, 0.25 µm film thickness). Helium was used as the carrier gas at a constant flow rate of 1.0 mL/min.

The injector temperature was set to 280 °C, with a split ratio of 5:1. The temperature program was as follows: initial temperature of 40 °C held for 1 minute, then ramped at 8 °C/min to 310 °C, and held for 7.15 minutes. Mass spectrometric detection was carried out using electron ionization (EI) at 70 eV. Data acquisition was performed in SCAN mode over a mass range of m/z 30–600. Compound identification was based on comparison with mass

spectra from the National Institute of Standards and Technology (NIST) library, version 14.0.

Figure S4 shows the chromatogram obtained from the analyzed sample, while Table S1 presents the identified secondary metabolites.

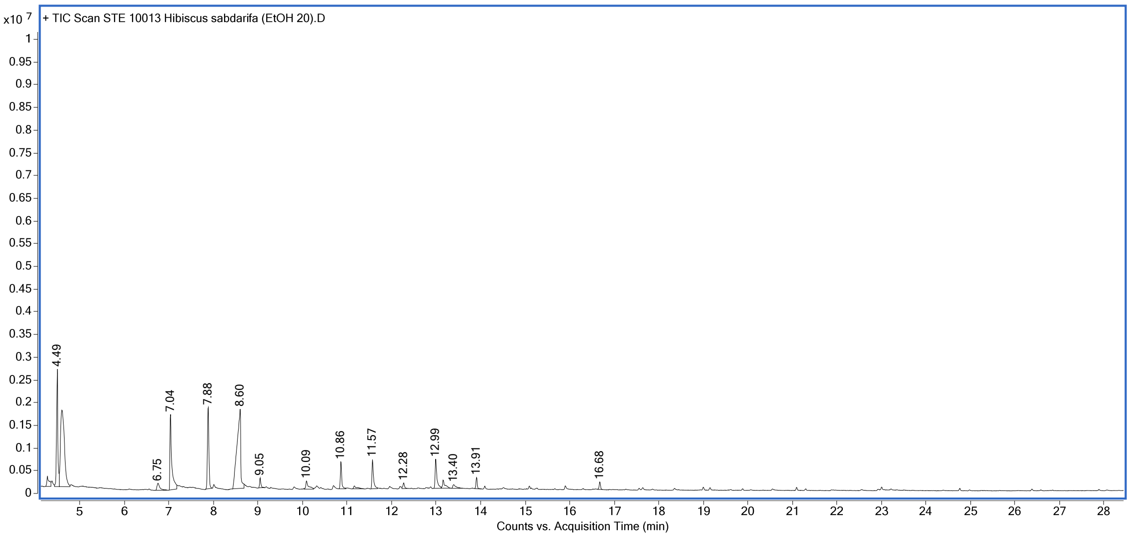

__Figure S4__ Chromatographic profile of the EHHs

Table S1 Identified compounds in the EHHs

Retention time (min)	Compound	Match	Area	% Area	Structure
4.27	Not identified		805807.13	1.80	
4.49	Acetic acid	99.30	5359458.21	11.98	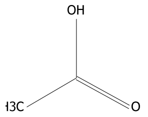
4.61	Cyclotrisiloxane, hexamethyl-	98.00	10347189.33	23.12	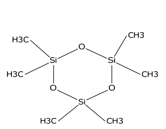
6.75	Not identified		693399.59	1.55	

7.04	2(5H)-Furanone	99.00	4723026.25	10.56	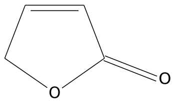
7.88	Cyclotetrasiloxane, octamethyl-	97.10	4098976.86	9.16	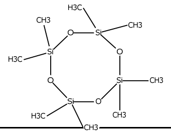
8.60	Propanoic acid	86.10	10249111.96	22.91	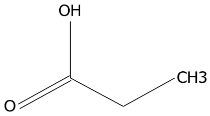
9.05	Not identified		430853.08	0.96	
10.09	Not identified		759910.96	1.70	
10.86	Cyclopentasiloxane , decamethyl-	97.90	1300849.39	2.91	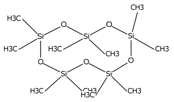
11.16	Not identified		339370.24	0.76	
11.57	Not identified		1559165.13	3.48	
12.28	Not identified		328178.31	0.73	
12.99	Not identified		1801693.94	4.03	
13.16	Not identified		693037.48	1.55	
13.40	Not identified		395118.28	0.88	

13.91	Cyclohexasiloxane, dodecamethyl-	98.10	506435.66	1.13	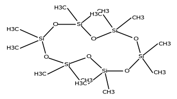
16.68	Cycloheptasiloxane, tetradecamethyl-	90.50	354190.14	0.79	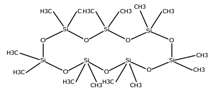
